# Experimental Assessment of the Fire Resistance Mechanisms of Timber–Steel Composites

**DOI:** 10.3390/ma12234003

**Published:** 2019-12-02

**Authors:** Truong Di Ha Le, Meng-Ting Tsai

**Affiliations:** Department of Architecture, National Taiwan University of Science and Technology, Taipei 10607, Taiwan; diha87@gmail.com

**Keywords:** timber–steel composite, fire resistance, Eurocode 5, dowel connection, charring rate

## Abstract

Hybrid structures known as timber–steel composites (TSCs) have been extensively studied due to their potential use as alternative construction materials that can satisfy demands related to sustainability. In addition to load capacity, fire resistance is a major consideration regarding the extensive use of TSCs. In this study, 12 specimens were tested using a glulam timber material covering cold-formed steel at the center. Specifically, the TSCs were fabricated from two timber blocks and an I-shaped steel core assembled using dowels or glue as a major structure. In order to use additional timber as a fire protection layer to protect a major structure by its charcoal produced after being burned, an additional timber with 5 cm in thickness was used to cover the major structure. The 1-h fire testing of TSC following the ISO 834-1 standard was applied, in order to achieve the potential application for a 4-story timber building. The results showed that temperatures at the steel flange increased by more than 300 °C for the final 5 min in 10 out of the 12 TSC specimens, indicating that the fire protection provided by the timber structure was not sufficient. The charcoal layer surpassing the extra timber was originally set and entered the steel structure of the TSC, which was expected to retain its physical qualities after a fire. Methods for evaluating the charring properties, based on the conventional method for wood and the standard specification set by Eurocode 5, were used to assess the structural degradation of TSCs. The conventional assessments showed a divergence from the actual performance of TSCs. Such variations demonstrated the limitations of models for conventional wood in assessing the structure of a TSC. A realistic assessment was conducted to expand knowledge related to this composite under destructive processes and provide fire reference values for the practical implementation of TSCs.

## 1. Introduction

A combination of two or more structural materials is known as a hybrid structure. In construction, hybrid structures are used for numerous applications. The outstanding performance of this material means these structures have the potential to protect against adverse factors in external environments [1,2]. The most common composite made from the mixture of constituent materials is reinforced concrete. Within this finished structure, steel plays a key role in tensile resistance. Concrete offers the most benefits in terms of compression and serves as a fire protection layer. From a sustainable point of view, a timber building is considered a renewable, recyclable material, especially the advanced development of engineered wood. Unlike a conventional light wooden building, engineered wood enhances the scale of timber building from lower story to higher. Moreover, the prefabrication of engineered wood allows the wood to be combined with other material efficiently, such as steel, by glued or dowel connection, in order to achieve the thermal [3], acoustic [4], or even structural performance [5,6]. Recent studies on timber and steel components (TSCs) have introduced a competitive strength versus reinforced concrete relationship in an age where the demand for sustainable practices is increasing [7]. These hybrid structures were available early in Europe, with wooden blocks used as reinforcement to resist the torsion, buckling, and curvature of thin steel sheets [8,9]. In Japan, the hybrid method has been available since steel first played a major role in load-bearing at the structure center with timber acting as an insulating factor [10]. A recent study in Taiwan on the cooperative attributes of individual components was carried out using steel as reinforcement to renovate common wooden structures. Beams with a cross-sectional dimension of 98 × 146 mm^2^ comprising two blocks of wood and an I-shape steel core were combined into one single beam using different dowels [11], as shown in Figure 3. The initial bearing capacity of the TSC beam structure was analyzed. However, to apply this structure in practice, information on its performance under the influence of fire is necessary; in particular, variations in behavior under different assembly conditions should be sufficiently explicit.

The design of the TSC beam has a considerable influence on stiffness once the shape of steel is optimized [12,13]. Although the theory of shape factors indicates that the I-shape of steel is crucial for evaluating the initial strength of the structure, the complexity of the shape factor also has a considerable effect on fire resistance [14,15]. Consequently, it is difficult to estimate the heat transfer within a heterogeneous structure. The Timber Engineering Council, using a design by the Vienna University of Technology, the Center for Fire Safety Science in Tokyo, developed a scale TSC model [16]. The insulation capacity of a 6 cm additional timber layer of TSC is assessed using 90-min fire experiments. By assessing the safety level of the point referring to the steel section, thermal transmission at the upper and lower flanges, inside and outside of the steel component, were determined. The heat transfer inside the steel component, which has implications for temperature safety, could therefore be evaluated. Although the temperature of the steel component was measured and simulated, the correlation between the two composite materials was not elucidated. In terms of practical applications, a predicted model of the cross-section of an efficient TSC is not yet available for use. 

Charring is the deterioration of timber from unburned wood to the pyrolysis zone and char layer. The distance between the char line, which is located at the 300 °C isotherm, and the outer surface of the original member is known as the char depth (CD). The rate at which wood changes to char is known as the charring rate (CR) and is measured in millimeters per minute. CD and CR are the most important indexes used to evaluate the fire resistance of combusted materials such as timber [17,18,19,20,21]. Such indexes govern the fire-related building codes and the safety level of the structure should be evaluated to avoid fire damage [22,23,24]. A Eurocode 5 (EU5) standard has been introduced to evaluate the charcoal layer of timber based on a purely linear material [25,26]. In the design of TSCs, timber functions as a fireproof layer that covers the main load-bearing component by stopping self-charring without the application of an incombustible material to the structure [27]. Once the wood layer is threatened, heat transfer into the steel increases the temperature of the adjacent wood, corrupting the component from the inside. Surface recession is therefore dysmorphic. The assessment of the irregular char layer becomes more complicated than those that follow linear-analytical standards; this should be addressed in studies. A recent study on TSCs explored the behavior of these structures in a fire lasting 10 minutes [28]. Within this short period, the char shape and rounding method indicated that char rate growth occurred relatively isotopically. The study only validated a simple estimation when the additional timber layer was thick compared with the burning time, and there was no air gap to allow the fire to penetrate the steel layer during combustion. To describe TSC behavior accurately, this study analyzed an anisotropic model based on the interactive relationship between I shape and timber thickness. Timber properties and the fastener method were analyzed from a 1-h fire test. Two types of wood materials were chosen for their practical application, the major imported wood species, Douglas fir, which is widely used in the local construction sector is selected in this study, in order to compare the performance with domestic species cedar in Taiwan. The fire testing with different methods of assembly (comprising glue and bolts) was examined respectively.

### Literature Review

**Figure 1:**
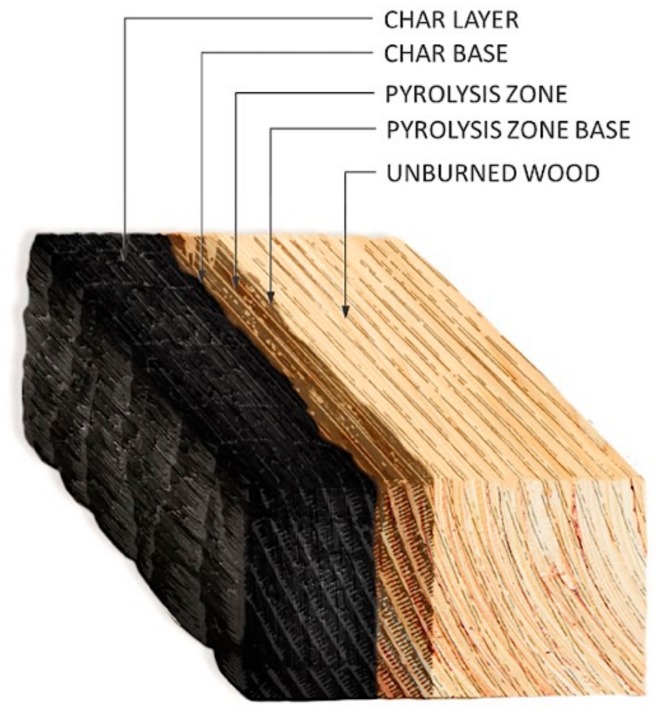
Degradation zone in a section of burnt wood.

As a consequence of the pyrolysis process, a charcoal layer was produced on the timber surface exposed to fire, as shown in Figure 1. Although the char layer did not contribute to mechanical force resistance, it played a substantial role in thermal insulation by protecting the inner part from fire caused by charring. Charcoal covered almost the entire surface of the specimen exposed to fire during the experiment. Martinka identified a method for predicting the loss of wood under fire, where the parameters of char layers become key factors for designing fire resistant wooden structures [29]. To determine the relationship between material loss and exposure time, White conducted a test on timbers that determined char layers produced in relation to wood density, moisture content, temperature, and fire-retardant chemicals [30,31,32]. The CR of solid wood samples tested primarily by ASTM E 119 or ISO 834-1 can be estimated [33,34]. The glulam specimens in this study were allowed to cool naturally inside the furnace before being cooled with water. According to CNS 12514, the CD and CR can be calculated as follows:

(1) $$C_{d\left(s\right)}=(S_{1}-S_{2})/2,$$

(2) $$CR_{\left(s\right)}=C_{d\left(s\right)}/t.$$

Another single-parameter model for fire resistance introduced by the American Forest and Paper Association shows the estimated CD as [33,35]:

(3) $$t=m_{1}x_{0}.$$

White also stated that the CR, which was conventionally considered to be 0.635 mm/min (1.5 in/h), was in fact 1.575 min/mm. Regression of all data was studied by White for different composite lumber products, producing $m_{1}$ at a rate of 1.53 min/mm. This can be converted to a CR of 0.654 mm/min. The average value collected during his experiment in 1992 was formulated as follows:

(4) $$t=CR.C_{d}^{1.23},$$

where $C_{d\left(s\right)}$ is the average charring depth of glulam lateral sides (mm), $S_{1}$ and $S_{2}\;$ the glulam widths before and after the fire exposure test, $CR_{\left(s\right)}$ is the average CR of glulam lateral sides, and *t* is the heating time in minutes.

**Figure 2:**
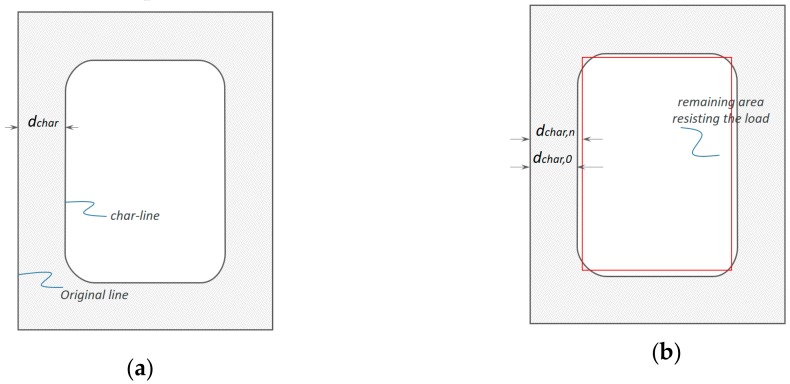
Concept for charring models. (**a**) Actual char depth (CD) $d_{char}$; (**b**) CD $d_{char,0}$ for one-dimensional charring and notional charring depth $d_{char,n}$.

EU5 provides different methods for calculating CR; there is a CR for one-dimensional charring and a notional CR for standard fire conditions [25,26,36,37]. This simple model uses the concept of CR as a function (Figure 2a) that is proportional to the destroyed structure in depth (mm) with the total time of heat exposure (min). When the width of the original section is greater than a certain value measured by $b_{min}$ (mm), the one-dimensional CR is applied. This is calculated as:

(5) $$b_{min}=\left\{\begin{array}{c} 2d_{char,0}+80\;\\ 8.15d_{char,0} \end{array}\right.for\;\begin{array}{c} \;d_{char,0}\geq 13\;\mathrm{mm}\\ d_{char,0}<13\;\mathrm{mm} \end{array}.$$

In this study, the thickness of each timber block in the TSC was smaller than $b_{min}$; hence, notional CRs were applied. The effect of the air gap caused by glue or dowel connections was also considered, the magnitude of which is influenced by the effect of corner rounding and fissures. The TSC composite, considered a notional CR design with a cross-section, was greater than $b_{min}$. The CR for notional charring (Figure 2b) was assumed to be constant over time. The design charring depth should therefore be calculated as:

(6) $$d_{char,n}=\beta_{n}t.$$

$d_{char,n}$: the notional design charring depth, which incorporates the effect of corner rounding;

$\beta_{n}$: the notional design CR, the magnitude of which is influenced by the effect of corner rounding and fissures;

t: The time of fire exposure.

## 2. Materials and Experiments

### 2.1. Materials and Major Structure

**Figure 3:**
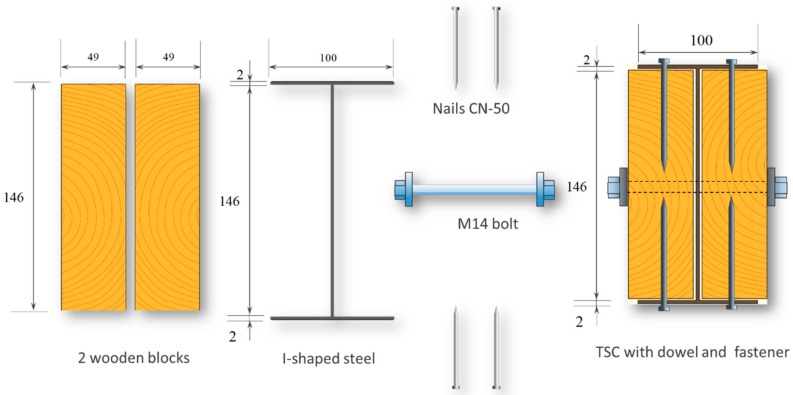
Major structure of the timber–steel composite (TSC) (unit: mm).

**Figure 4:**
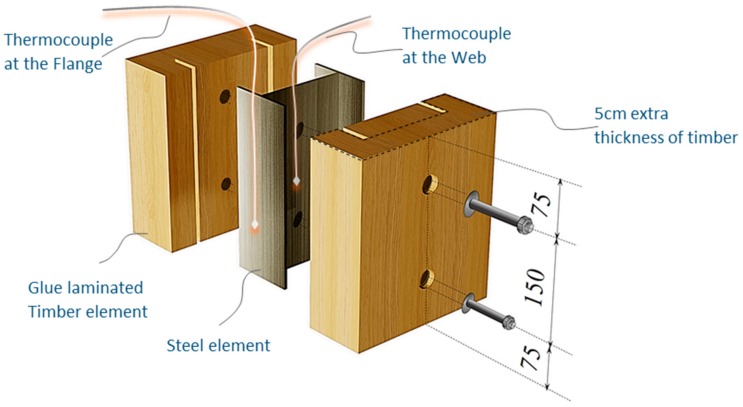
TSC specimen with dowel connection equipped with thermocouples (unit: mm).

Douglas fir (Pinaceae genus) with a density of 537.63 kg/m^3^ and cedar (Cedrus) with a density of 442.97 kg/m^3^ were selected to compare differences between imported and domestic wood. CNS14630 standards in Taiwan were applied to control the water saturation of wood to less than 15% after kiln drying. After drying, two types of glued laminated timber (glulam) were used; these were prefabricated. The two wooden blocks are 300 mm high with a cross-section of each timber measured 49 × 146 mm^2^, as illustrated in Figure 3. Based on CNS6183, the regulations on the size and shape standard of cold-formed lightweight steel in Taiwan, the cold-formed steel with elastic modulus of 203,000 N/mm^2^ was used. The dimensions of the I-shaped steel and steel plate were 150 × 100 mm^2^ and 2 × 2 mm^2^, respectively, and both materials were 300 mm in height. M14 bolts and glue were used to examine the fire resistance effect of different connections. The TSC illustrated in Figure 3 was considered the major structure following the previous study [6,11]. In this study, in order to use additional timber as a fire resistance layer to protect major structure, an additional timber with 5 cm in thickness was used to cover the major structure (Figure 4). 

### 2.2. Specimens

The TSC with fire protection added is illustrated in Figure 4, and the specimen types are summarized in Table 1. The details of these specimens are as follows. Type DB is a TSC with glue-laminated Douglas fir connected to I-shaped steel with a dowel. Type DO is a TSC with glue-laminated Douglas fir connected to I-shaped steel by glue. Type CB and CO are TSCs with glue-laminated cedar bolted with I-shaped steel and glued with I-shaped steel, respectively. The spacing of the bolts was 150 mm: this was the spacing used in a study of built-up beams and is the typical spacing used for timber wall connections in Taiwan [35]. When the TSC member is exposed to fire, causing heat transmission, the dowel connection at the web transfers heat differently to the glue connection and hence must be examined. Three specimens for each type of TSC (DB, DO, CB, and CO) were tested (12 specimens overall). Each specimen type was tested according to the ISO 834-1 standard [38].

**Figure 5:**
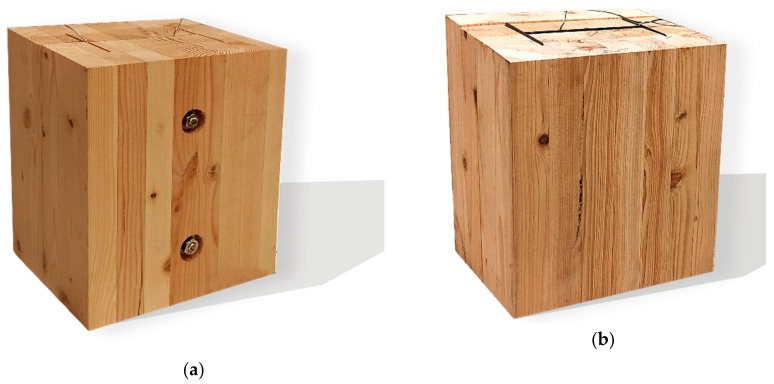
TSC specimens: (**a**) dowel connection and (**b**) glue connection.

The specimens shown in Figure 5 were placed into the furnace (Kuo Ming Refractory Industrial Co., Ltd, Taiwan) for testing. The upper part of the section was covered by rock wool for fire protection. Two thermocouples (Yi-Tai System Technology Co., Ltd, Taiwan) were installed inside each set of TSCs to monitor the temperature inside the TSC column, especially the steel member. One was placed on the flange of the steel member and the other on the web of the steel member, as shown in Figure 4 and Table 1. The furnace was also equipped with thermocouples to record the environmental temperature.

### 2.3. Testing Machine

**Figure 6:**
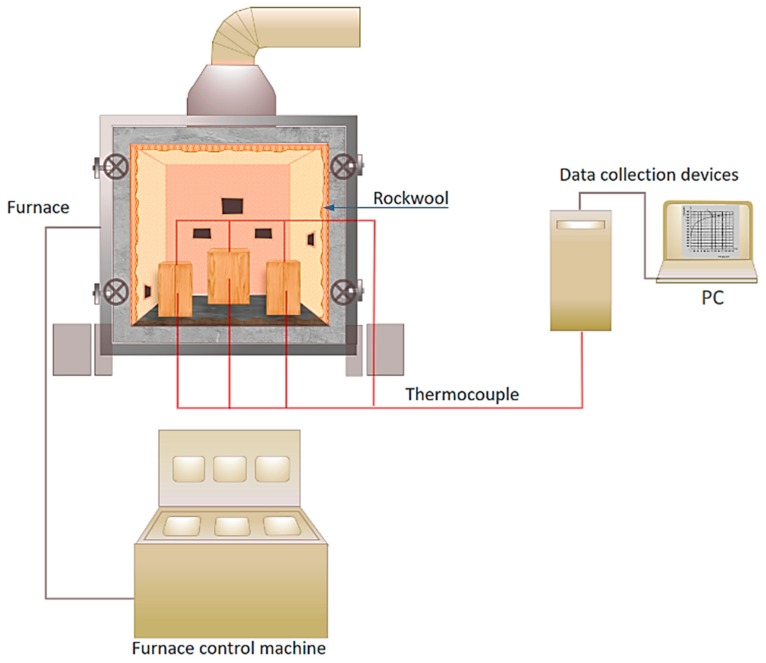
Furnace and test setup.

**Figure 7:**
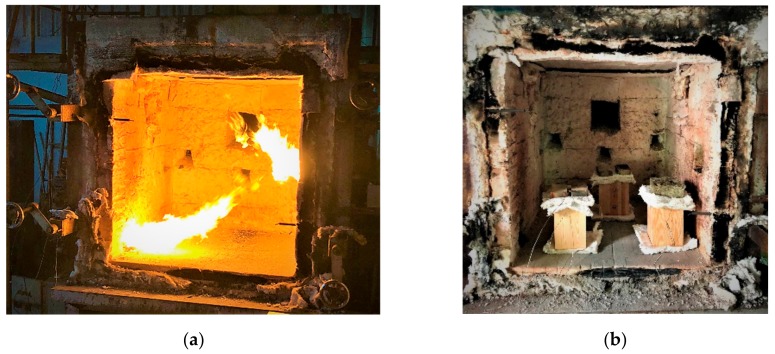
(**a**) location of fire; (**b**) arrangement of test specimens.

The width, length, and depth of the inner part of the furnace machine were all 120 cm. Each TSC specimen was installed with two thermocouples, which were used to investigate the difference in temperature between the inside and outside of the steel core at the web and flange of the I-shaped steel (Figure 6). The ignition was executed through creating two large holes (Figure 7a), which may have affected specimens, depending on their position in relation to the holes (Figure 7b). The temperature in the furnace was controlled by a preprogrammed machine that conformed to ISO 834-1 requirements. The multidigital device recorded and converted data into a digitized format with 4-s time steps.

According to ISO 834-1, the top part of trunked columns must be covered by a noncombustible material. Only four rest surfaces were vulnerable to fire damage. There is no risk of fire spreading as a result of thermal radiation when the unexposed surface temperature is below 300 °C. A test on the fire behavior of the TSC beams was conducted in accordance with ISO 834-1. Furnace productivity was recorded by examining the relationship between temperature (t) and time (min); gas and ignition were controlled by a central supply system. Following ISO 834-1 and ISO 834-6, the furnace temperature reached approximately 1000 °C within 60 min of burning, as represented in Equation (7):

(7) $$T=20+345\times {log}_{10}{\left(8t\;+\;1\right)}_{.}$$

## 3. Methodology

### 3.1. Charring Depth Based on Eurocode 5

Although North American studies have primarily been conducted on a specified number of available wood species, as discussed in the previous section, such models involve the use of essential parameters that are based on wood properties, including density, percentage of moisture, chemical composition, and permeability. To identify the fire resistance of a composite contribution, a TSC model may not be sufficient to navigate on a timber parameter alone. Although the CR may depend on wood properties and increase with time, based on the EU5 method, it is constant in practice. Therefore, the applicability of the simplified EU5 method was assessed and modified to adapt a hybrid attribute for this structure. 

Given that the timber-based materials in the experiment were softwood, unprotected throughout fire exposure, and in accordance with EU5, the design CRs $\beta_{0}$ and $\beta_{n}$ were used (Table 2). For cross-sections calculated using one-dimensional design CRs, the radius of the corner rounding was taken to be equal to the charring depth $d_{char,n}$, based on the actual CD measured in the experiment. Design CRs for solid hardwoods, except beech, with characteristic densities between 290 and 450 kg/m^3^, were obtained by linear interpolation and ranged between 0.65 and 0.7 mm/min. The CR of glued laminated timbers, the densities of which were greater than 290 kg/m^3^, yielded a notional CR of 0.7 mm/min. 

According to EU5, an effective cross-section can be calculated by reducing the initial cross-section by the effective CD $d_{ef}$ (Figure 9). On the basis of Equation (6), $d_{ef}$ allows for an additional thickness level through the use of a reduced cross-section method that specifies applying an additional section in the structure to improve safety:

(8) $$d_{ef}=d_{char,n}+k_{0}d_{0},$$

$d_{0}$: 7 mm;

$d_{char,n}$: Determined according to expression (1.1);

$k_{0}$: Coefficient, $k_{0}$*= 1* because t > 20 min.

### 3.2. Charring Depth Modified by the Average Charring Area Method

**Figure 8:**
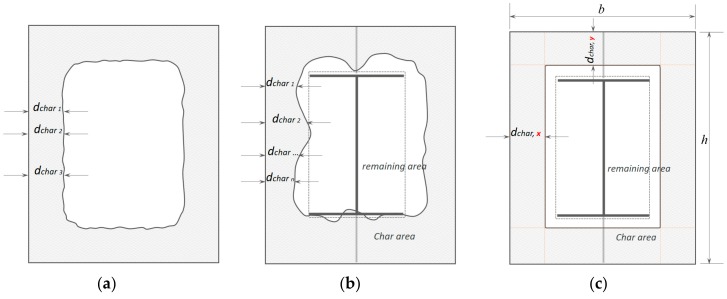
Concept for charring calculation. (**a**) conventional CD model; (**b**) char area model observed from testing; (**c**) average char area model for CD calculation based on the char area method.

For the conventional char rate calculation model [39,40], CD was taken as an average of the char layer thickness, which was three measurements on the timber thickness after the charcoal layer was removed, as shown in Figure 8a. The TSC char line, however, was irregular, as shown in Figure 8b. Determining the average CD under the conventional model requires the use of all the measurements on the *x*-axis and *y*-axis. A conventional measuring approach thus limits CD accuracy.

To avoid missing other $d_{char}$ values a TSC may produce, the average method that employs area measurement was adapted (Figure 8c). If the same char area $A_{char}$ obtained from the experiments shown in Figure 8b is used, the average charring area model is based on the principle that all CDs in the *x*- and *y*-directions are expected to be uniform. The average charring area model tends to redistribute the char area linearly, allowing use of the same predicted char line as the conventional technique. This is based on the following quadratic equation:

(9) $$4d_{char,x}^{2}-2\left(b+h\right)d_{char,y}+\;A_{char}=0,$$

where $d_{char,x}$ is the CD along the x axis; $d_{char,y\;}$ is the CD along the y axis ($d_{char,x}$ is expected to be equal to $d_{char,y\;}$); *b* is the width of TSC; *h* is the height of TSC; and $A_{char}$ is the char area. The average CD is a positive solution of the quadratic Equation (9), which is then written as:

(10) $$d_{char,average}=\;d_{char,x}=d_{char,y}.$$

According to Equation (10), CD is derived from the charred area that covers the CD equally along the *x*- and *y*-axes of the cross-section. This limits the errors that might occur by using the conventional approach of taking manual measurements. The average charring area model can then be verified using the experimental results. The specified reduced cross-section model was included in the evaluation to validate the behavior of elements under fire in a condition given by EU5. The models were then compared with the experimental results. The assessment of the average charring area model was based on comparing the $d_{ef}$ specified model by EU5 with the highest $d_{char}$ value of the maximum charring depth model, which is described in the following section.

### 3.3. Maximum Charring Depth

**Figure 9:**
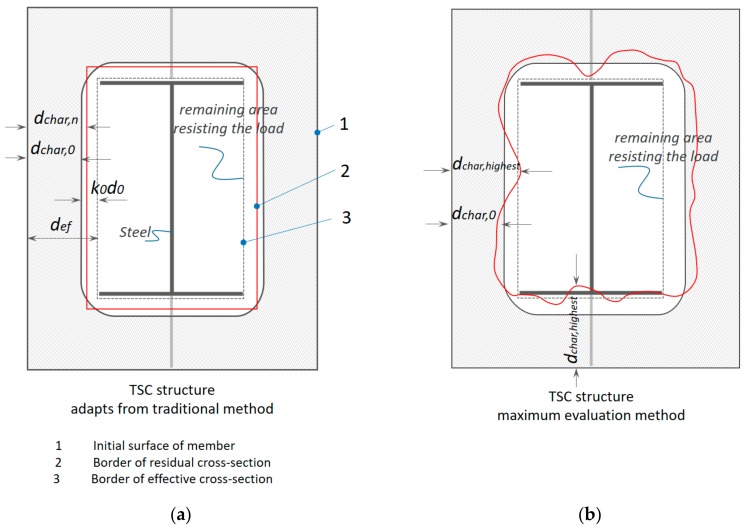
Concept for determining the charring line. (**a**) average charring area model adapted from EU5; (**b**) maximum charring depth model.

The model evaluates the security of the component based on the maximum char rate and is therefore known as the maximum experimental evaluation model. It was assumed that the material close to the char line (Figure 9a) in the layer of thickness $k_{0}d_{0}$ (included in Equation (8)) has zero strength and stiffness, whereas the strength and stiffness of the remaining cross-section was assumed to be unchanged. Using the maximum charring depth model, the CD result was then compared with the $d_{ef}$ given by EU5 Equation (8). Adapting the maximum CD from this model, the max CR $\beta_{TSC\left(max\right)}$ was then calculated based on Equation (6) specified by EU5, (Figure 9b). The results of the comparison are described later in the discussion to assess the prerequisite that the actual structure satisfies the criteria under fire. If one of the criteria is not satisfied, the actual structure is considered dangerous:

(11) $$d_{char,max}=\beta_{TSC\left(max\right)}t.$$

$d_{char,max}$: The highest design CD, which incorporates t;

$\beta_{TSC\left(max\right)}$: The highest design CR, the magnitude of which includes the effect of corner rounding and fissures;

t: The time of fire exposure.

## 4. Results

### 4.1. Experimental Results

**Figure 10:**
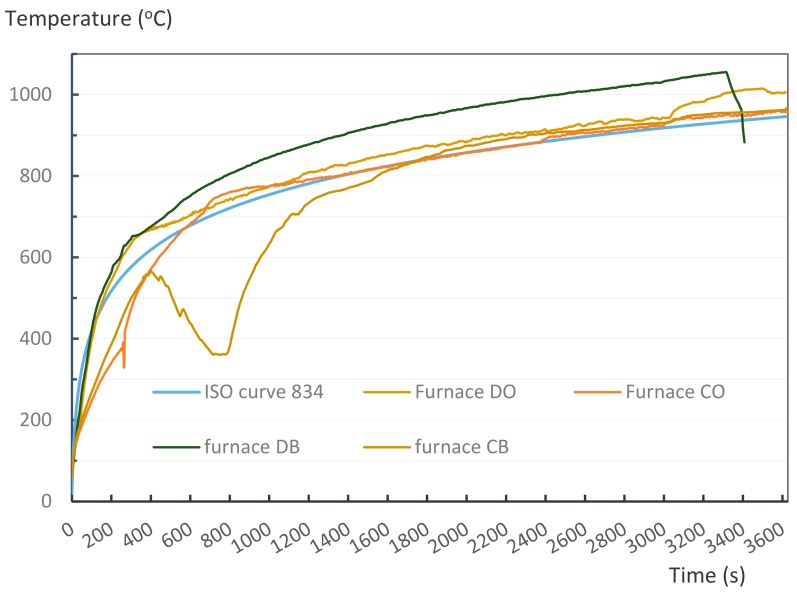
Grade of experimental furnace heating and ISO 834 curve.

The furnace temperature for the four sets of experiments is presented in Figure 10. The furnace temperature changed each time the experiment was conducted and the supplied heat was unstable depending on the gas supply of the system. Due to the quality of the machine, the actual conditions of the furnace were not controlled precisely; therefore, errors occurred. The fire temperatures were slightly different to the ISO curve within an hour of burning. For the CB combustion experiment, the furnace temperature did not follow the ISO curve from 400 s and dimmed within 10 min up until 1000 s. The furnace temperature of the DB set was relatively higher than that of the other sets at approximately 100 °C. However, these differences did not significantly affect the results observed in the samples. The disparity of the charring rate and the temperature of these samples show comparable results, with no more than 8% difference compared to other samples.

#### 4.1.1. Temperature Tracking by Thermocouple

**Figure 11:**
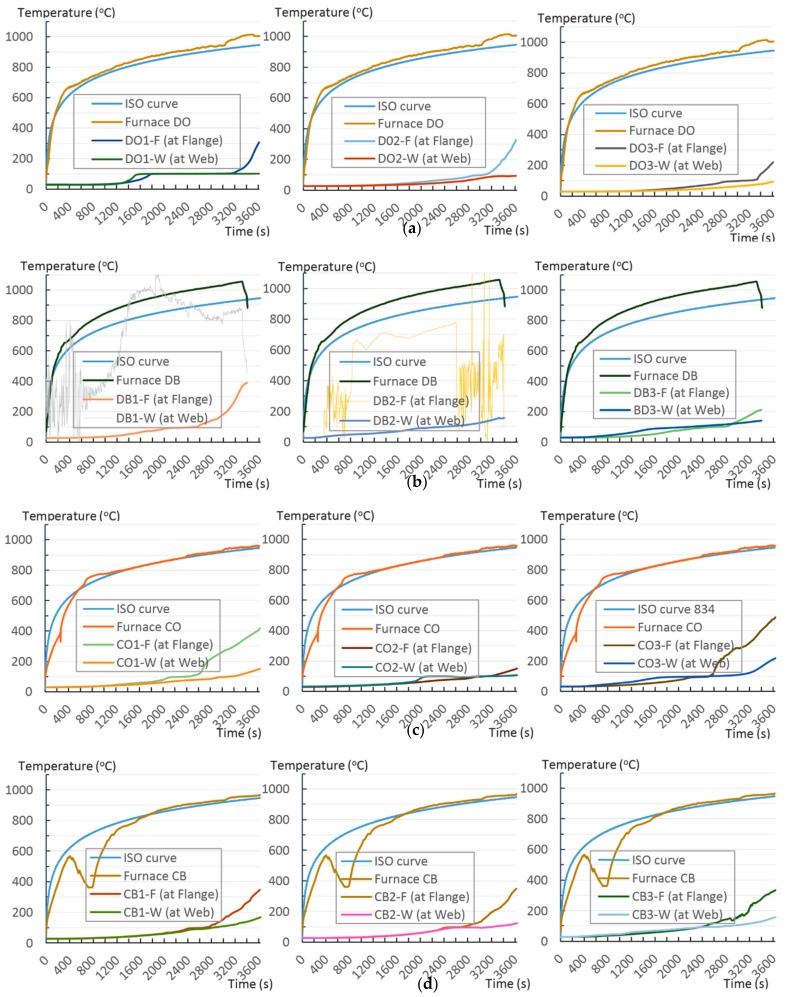
Specimen heating temperatures. (**a**) Douglas fir TSC assembled using glue; (**b**) Douglas fir TSC assembled using dowels; (**c**) cedar TSC assembled using glue; (**d**) cedar TSC assembled using dowels.

According to EU5, thermal radiation below 300 °C is safe for an unexposed surface, which means there is no risk of fire spreading when the temperature recorded on the indicated surfaces is less than 300°C. During the test, 8 out of 12 specimens exceeded the safe limit at which timber will be turned into char by the heat. At the end of the heat chart, the temperature at the flanges is approximately twice as high as the temperature at the webs, as shown in Figure 11. Furthermore, some TSCs exceeded the expected limits, such as CO3 and CO1 (486 °C and 417 °C, Figure 11c). Within the first 20 min of a 1-h fire experiment, the temperature measured at the flange and the web of the TSC appeared to merge. This means that, in a short time, the effect of fire on the measured points was negligible inside the TSCs, where the temperatures were below 30 °C. After this point, the temperature difference between the flange and the web of the TSC structure began to increase (Figure 11a,b,d). The temperature in the TSC web, which was isolated 10 cm from fire exposure by timber, increased slightly but remained at a safe margin below 200 °C. By contrast, the temperature at the flange, which was 5 cm from fire exposure by timber, dramatically increased. The increase in flange temperature depended on the wood material as well as the methods of connection; further details are provided in the following section. 

The unsatisfactory heat that was recorded is described briefly as follows. Specimens DB1, CO1, and CO3 (Figure 11b,c) were found to have a flange temperature higher than 300 °C from 55 min onward. All three specimens in the same timber set of cedar samples with bolts, namely CB1, CB2, and CB3 (Figure 11d), had a recorded temperature above 300 °C after 58 min. Two of the specimens in the timber set of Douglas fir samples fastened by glue (Figure 11a) showed a slight elevation of 300 °C after 58 and 57 min. The unexpected performance of DB1 at the web and DB2 at the flange position occurred approximately 5 min after fire started, which was believed to be related to the failure of the thermocouple. However, the values obtained from thermocouples from the rest location at DB1, DB2, and each of thermocouples from DB3 were kept for evaluation as they still play a vital role in the observation and examination results. Hence, it was assumed that the independent measurements within three specimens are meant to describe the behavior of a TSC under the completed examine.

#### 4.1.2. Failure Modes

According to EU5, CD is defined as the distance between the outer surface of the original member and the position of the char line for pure wood. The distance between the char line, located at the 300 °C isotherm, and the outer surface of the original member is defined as the CD. The intensity of the black color denotes the thickness of the coal layer. The lighter area represents the pyrolysis zone of the wood. The experiments measured the area of wood charred on the section using the ASTM method. The specimen was removed from the furnace after a 1-h ISO fire. Data for all samples were recorded immediately after such samples were removed from the oven and cooled, as shown in Table 3. 

The deterioration of the wood was measured based on the area of the char layer. The char area accounted for 47.4%–66.2% of the wood area and char amounts are recorded in Table 4. Because this study has not considered the load capacity testing of the beam after burning, it was essential to analyze the structural degradation based on the char map. Wood degradation in the web reduced the antibuckling function, whereas the degradation of wood at the flange affected the temperature and physical properties of steel. The structure is considered unsafe whether or not the char line overstepped the TSC line at the flange or the web. The char line of these maps and the TSC line, which were 5 cm from the original line of the structure, were then compared. Percentage of char over the total area of TSC measured as $A_{char}/A_{TSC}$ (%) and the percentage of char over the observed extra timber area $A_{char}/A_{extra\;layer\;}$(%) could not fully explain the behavior of the material due to TSC’s heterogeneity. Therefore, the char map is further explained in the discussion section. The visible char map reveals the threatened structural location; thus, the quality of the remaining structure can be evaluated. 

The two levels of char assessment observed from the experiment are defined in Table 5 in columns 3 and 4 as safe or dangerous if $d_{char,max}$ remains within 5 cm or exceeds this major benchmark, respectively. The quality of the structure also depended on the highest temperature of the steel after 1 h of burning (Table 4). According to EU5, thermal radiation below 300 °C is safe for unexposed surfaces, which means there is no risk of fire spreading. The evaluation of failure is based on both char failure and failure in temperature criteria. Therefore, these temperatures (second column) along with the criteria of the char line were used to assess the quality of the structure. Overall, 75% of the samples failed according to thermal property requirements. Furthermore, 50% and 58% of samples were found to be unsatisfactory due to deterioration in the flanges and webs, respectively. During the test, 9 out of 12 specimens exceeded the safety limit, as indicated in Table 5.

### 4.2. Comparison of Different Assessment Models

#### 4.2.1. Charring Depth

The char layer was removed and the area of the charcoal layer obtained by taking the remaining area from the original TSC area. By measuring the remaining areas of the timber elements through a method described earlier, the CD was collected. The percentage of charcoal area was also determined (Table 4). The average charcoal thickness, determined using an average charring area model, was approximately 30.48 to 46.32 cm. 

The average charring area model shows high agreement with the EU5 model for four types of TSC with a difference ratio of less than 7%, as shown in Table 6. The difference ratio between these two models was 3.3%, 8%, and 4.3% for DB, CO, and CB, respectively. DO yielded the highest difference ratios of 23%, which is lower than in the specified model. Only DB yielded a CD value higher than the specified value; the others had lower values in which the calculated CD remained close to the standard value. However, because the actual maps of the charcoal layer were considered, the conventional method, recorded from the average CD, disagreed with the practical requirements in terms of the final shape of the TSC. More than 74% of the experimental specimens exceeded the allowable limit of 5 cm of degradation. Experimental simulation modeling, which was used to investigate the maximum damage to TSCs, reflected the influence of fire-related factors on the TSCs, as follows. Most of the maximum CD measurements exceeded the TSC line, which was larger than 50 mm. Only two specimens, DO2 and DO3, were below this allowable level (44 and 43 mm, respectively), and two specimens, CO2 and DB3, reached this level (50 mm). In general, the different ratios between the maximum char model and the EU5 model were approximately 24.8% and 31.8% compared with average char area model, respectively.

#### 4.2.2. Charring Rate

**Figure 12:**
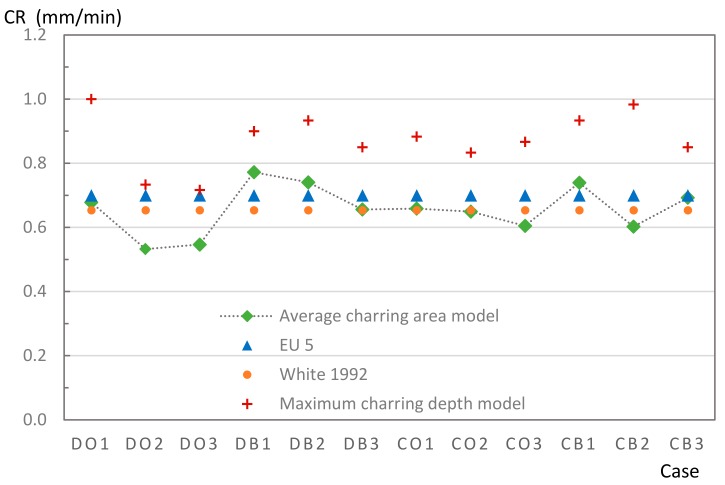
Experimental results and comparison of CR models.

To determine the CR, the average method, which was calculated according to Equation (10), was applied. The CR obtained from the average method, experimental method, and the designed CR given in EU5 were then compared, as shown in Figure 12. The average CR of all models obtained from the conventional model was highly similar to the EU5 model. There were two patterns that approximately matched the CR given by EU5 (0.7 mm/min), seven patterns were below this level, and three were slightly higher. The other CR introduced by White was also included in the comparison to examine the reliability of study modeling, as described in Section 3.1. White’s model also showed strong agreement with the model given by EU5 at 0.65 mm/min and 0.7 mm/min. The model also strongly agreed with the average char area model, with only a small ratio difference observed (2.3%). However, the maximum CR values for the experimental model were high at 0.87 mm/min. The CB was highest with 1 mm/min, although even the lowest CR was higher than the CR given by EU5. For the group of CB specimens, the CR was the highest, on average, of all the specimens.

### 4.3. Evaluation of Effective Cross-Sections Based on Different Methods

To examine the validity of the models, a parameter based on the EU5 designation was included in the assessment. Evaluation criteria based on the depth of the charcoal layer were given by EU5 as an effective cross-section. According to Equation (8), as shown in Section 3.2, allowable charcoal layers *d_ef_* were compared according to an effective CD obtained by using the average method, maximum charring method, and CD given by EU5. The effective cross-section method provides a coal thickness that allows wood-based components to achieve efficiency within 1 h of burning. On the basis of this method, the allowed CD was 49 mm. All these reference values were compared with the experimental model, shown in Table 7, to determine whether the thickness requirements were satisfied. The results showed that only two samples, those of Douglas fir with glue connections (type DO), satisfied these requirements. The test specimens for this type comprised only group that conformed to the cross-section criteria for pure wood. The CDs of the remaining groups were higher than the standard CD at 8.8%, 6.1% and 12.3% for DB, CO, and CB, respectively. 

## 5. Discussion

### 5.1. Effect of Timber Material on TSC

Numerous studies have found an independent relationship between wood density and fire resistance. Because cedar wood has a lower specific gravity than Douglas fir, fire resistance is negligible. In this study, the wood had a specific volume that gives it a higher specific density, indicating opportunities for greater fire resistance. The temperatures at the flanges of the TSC measured for the Douglas fir were lower than that for cedar. However, within the scope of this paper, the data on the properties of wood were not sufficient for clarification; therefore, further verification on timber categories is required.

**Figure 13:**
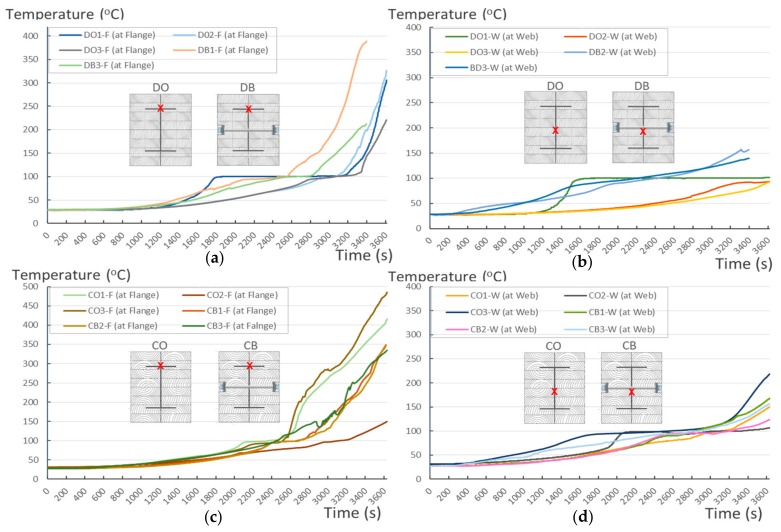
Thermal record of (**a**) all Douglas fir TSCs at flanges; (**b**) all Douglas fir TSCs at webs; (**c**) all cedar TSCs at flanges; and (**d**) all cedar TSCs at webs.

This section analyses the effect of timber material on TSC based on the test results obtained for a group of the same timber type, namely Douglas fir (DO, DB; Figure 13a,b) and cedar (CO, CB; Figure 13c,d). The furnace temperature was higher when Douglas fir was burned, although the results show that the temperature at the flange of the Douglas fir samples was still lower than the highest temperature of cedar flanges. Comparing the highest temperatures at the flange of the TSC group indicated that TSC beams with Douglas fir (Figure 13a) had a temperature that was approximately 100 °C lower than those of cedar (Figure 13c). The temperature gaps measured from the webs differed by approximately 50 °C; it was highest at 180 °C and 220 °C for type DB2 and CO3, and second-highest at 100 °C and 150 °C for DO1 and CO2, respectively (Figure 13b,d). At the end of the chart, the heat of the web in the cedar group had a sudden increase in the last 100 s (Figure 13c,d). This is in contrast to the Douglas group, wherein the increase in heat remained steady until the end of the experimental period. On the basis of the highest temperatures obtained from the flanges, the final temperature of the cedar group was 22.3% higher than that for the Douglas fir group.

### 5.2. Effect of the Connection Method on TSC

**Figure 14:**
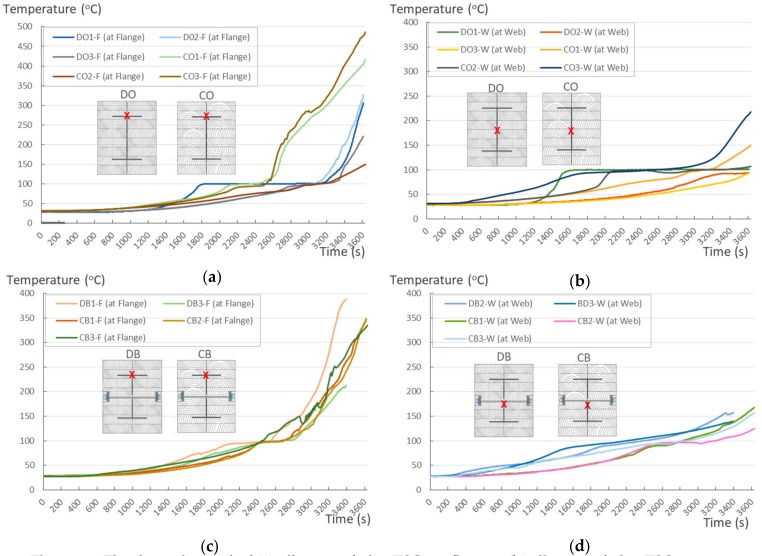
The thermal record of (**a**) all types of glue TSCs at flanges; (**b**) all types of glue TSCs at webs; (**c**) all types of dowel TSCs at flanges; and (**d**) all types of glue TSCs at webs.

The temperatures at the flange and web for dowel connection types rose steadily. The temperature measured from the flange of all TSC dowel connections showed a slight increase below 150 °C until 50 min, at which point a sudden increase to approximately 350 °C occurred (Figure 14c). The temperature trend at the flanges of both types of wood material in this dowel type had a tendency to merge (Figure 14c,d). The temperature recorded at the webs of the TSC for the dowel fastener had a similar trend, which was assumed to be a safe reaction to fire as it remained below 150 °C and increased steadily during 1 h of burning (Figure 14d). The performance of these thermal lines of glue connections exhibited sudden increases at 30, 43, and 53 min; these were recorded at the flanges of both Douglas and cedar samples (Figure 14a). The temperatures recorded at the webs of the glue method TSCs showed a sudden increase in temperature at the 25, 33, and 53 min. Notably, after a sudden increase, the temperature reached twice that of the original temperature (Figure 14b). The glue connection was expected to provide higher fire resistance by sealing air gaps in the TSC section and providing better heat leak resistance. In this case, a connection with glue was only reasonable for the first 30 min. In terms of keeping the temperature stable, this structure was not as efficient as that of the TSC dowel (Figure 14a–d). On the basis of the highest temperatures obtained from the flanges of the groups, the dowel connection group had a final temperature that was 2.5% higher than that of glue group.

### 5.3. Assessment of Anisotropic Model

For homogeneous wood materials, under standard fire exposure conditions, the CR is relatively constant. If the timber mass is sufficiently thick, after being subjected to a high CR, the depth of the char does not depend on the entire thickness of the timber. The corresponding measurements given by this method are as follows.

TSCs with Douglas fir attached using glue had the lowest CR among the four types of TSC materials under fire conditions (Table 8). This shows an effectiveness of almost 100% for pure wood. The values obtained for DB, CO, and CB were approximately 91%, 94%, and 88% of that effectiveness, respectively. Ignoring differences in furnace heat in each experiment, this visible difference can be explained by two factors, namely differences in the microstructure of wood and dowel style. Douglas fir, which is denser than cedar, may provide greater protection against ignition phenomena than cedar; furthermore, it may avoid or delay charring on the exposed surface. A glue connection with a higher efficiency fills air gaps, which helps protect the inner part from fire exposure.

The char map statistics in Table 9 explain the relationship between the temperature of the flanges and the char map. The TSC can be predicted to be safe or should be restructured under fire conditions. DO3 is an example of a successful TSC after burning occurs, as the char map maintains a safe position in relation to the TSC line; therefore, the measured flange temperature for this sample is predicted to be low. However, some specimens showed char map patterns that penetrated deeply into the TSC line, such as for CO1, CO3, or CB2, resulting in a critically high temperature in the flanges. Noticeably, DO1 had a considerably degraded char map at the web position, which represents a low flange temperature. In summary, linear model EU5, which presents an average CD, cannot fully explain the differences in the malformation of the actual anisotropic TSC properties shown in Table 8. The char map exhibits a nonlinear shape; hence, the maximum distance from the original outer line to the char line is considered. This is useful for estimating the required depth for the TSC structure during the desired combustion time. The CRs collected in this study can therefore be considered a reference value for future TSC material-related studies.

The average charring depth value of Douglas fir was 5.3% lower than that of cedar. The difference reaction for this figure between the dowel and glue method was 2.5%. The TSC group assembled by Douglas fir with a glue connection yielded a CR of 0.82 mm/min, while the rate given by a TSC group connected by a dowel was 0.89 mm/min. The CR obtained from cedar with a dowel connection gave the highest value (0.92 mm/min), while the value of this wood under glue was 0.86 mm/min.

However, when an average charring area model of CD is used, this concentrates purely on the thermal model that eliminates the geometrical complications. The combustion process of pure wood without the interference of any other materials, or with negligible secondary materials, is an isotropic model. The level of deterioration of TSC, which degrades wood and loses its ability to transfer heat leading to the destruction of steel, performed independently of the average charring area model. The fire resistance mechanism of the TSC occurred as an anisotropic model, so an average charring area model for the pure wood material could not address a TSC model. Due to the complex structure of TSCs, the air gap allowing the fire to penetrate deeply into the structure, as well as the dowel or glue connections during combustion, affected the double-sided interaction of temperature. Therefore, the coal map should be carefully taken into consideration. 

**Figure 15:**
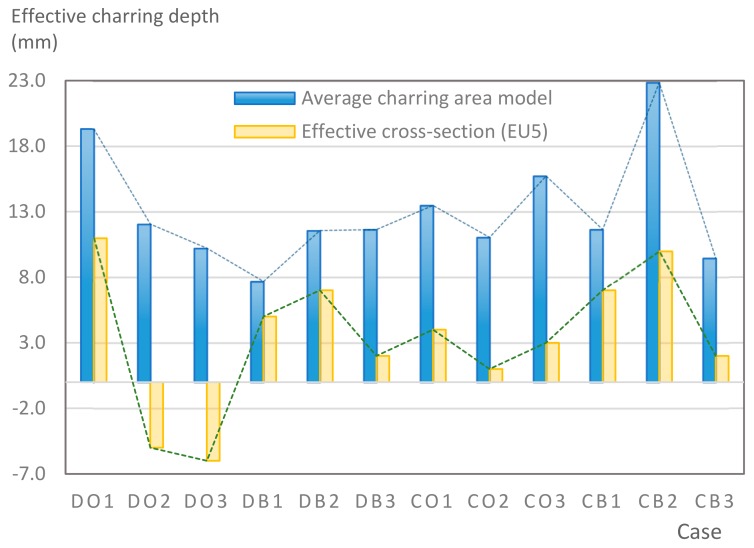
The additional timber thickness recommended by the methods.

From a design perspective, the deviation in CD can be determined by the methods presented in this study. Assuming that the efficiency of cross-sections is an intermediate factor, dotted lines reveal the additional timber thicknesses required of an effective TSC cross-section in a 1-h fire. Figure 15 shows the recommendation in the additional layer of timber for a TSC to achieve fire resistance based on the effective cross-section model by EU5 and the average charring area model. Negative values can be interpreted as zeros in the additional layer. Designers may need to increase the timber layer by 22% when they implement an effective cross-section using the EU5 method; they may even increase the layer by 45.7% for the average charring area method to ensure structural integrity.

### 5.4. Future Applications and Improvements

In this study, a maximized model adopted from the EU5 model was improved and used to evaluate the fire resistance of TSC beams assembled using different wood materials and connections. The results provide designers with a method for obtaining TSCs that are compliant with building codes under fire conditions, which providing at least 1-h fire resistance for the structural beam to maintain structural integrity in a four-story timber building. Furthermore, TSC beams are expected to be applied in the development of high-rise buildings (higher than four-story); thus, the higher level of safety requirement for longer fire resistance, for example 2-h, can be achieved by increasing the thickness of outer timber, which is CD. In that case, to satisfy the fire codes, further studies are required for more TSC’s aspect investigation. Although a scale model TSC can be determined, the determination of a full-scale beam is required, as is the prediction of the failure of wooden members when interacting with steel members. Further research must be conducted to clarify the behavior of the full-scale TSC beam under fire conditions.

## 6. Conclusions 

Experimental tests on differing wood properties and connections identified the aggregate behavior of TSC during a 1-h ISO fire test. The aim was to clarify factors related to the safety of a hybrid structure under fire conditions. Within the scope of the research, TSCs measuring 20 × 25 × 30 cm^3^ were assembled using local and import glulam timber materials with glue or dowel connections at the web. These TSCs had timber with an outer thickness of 5 cm. Overall, 67% of TSCs failed according to temperature safety requirements and 83% of TSCs failed according to charcoal layer degradation standards. However, the scale model used for the experiment limited the evaluation criteria as the bearing capacity of TSCs under fire conditions was not evaluated. A full-scale model with specimens must be tested to provide more precise information for future assessment. 

The most important finding in this study is as follows. The conventional approach for determining pure wood properties demonstrates the limitations in solving issues arising from the use of TSCs. The average charring area model, following the conventional method, and the model indicated by EU5, respectively, yielded 32% and 24.3% difference ratios from the actual results. Therefore, the thicknesses of timber elements in TSCs must increase from 12 to 15.7 mm to ensure fire resistance under a 1-hour burning condition. This is a reference value for the TSC hybrid timber–steel structure that was predicated using computation and experimental evaluations. A comparison of structural deterioration indicated that a Douglas fir TSC outperformed a cedar TSC (22% in terms of thermal capacity, 5% in CR). Compared with the dowel, a TSC using glue demonstrated a 7% higher efficiency regarding insulation capacity and 3% regarding CR. The difference in values can be attributed to the quality of wood characteristics and the rupture behavior of the dowel and glue based on their chemical and mechanical properties. The glue TSC structure has a more positive effect on fire resistance than a dowel TSC, whereas dowel TSCs can prevent the temperature from suddenly increasing inside the TSC. Douglas fir is helpful in preserving heat from the inside, while the glued TSC is better at preventing charcoal-related deterioration. Hence, designers should consider the most important factors depending on the purpose and use of the structure.

By determining the char rate according to Douglas fir or cedar and whether dowel or glue connections are used, a method for estimating an effectively reduced TSC cross-section for 1 h of burning has been introduced. Designers can refer to the resulting CRs to estimate the required thickness for a TSC during a certain burning time. However, dowel and glue TSCs must be studied further to improve the stability of the structure due to loss or separation that may occur during burning.

## Figures and Tables

**Table 1:** Cross-section of timber–steel composite (TSC) specimens.

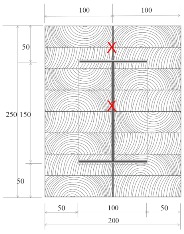	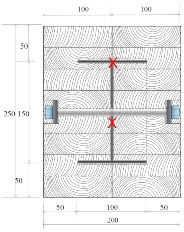	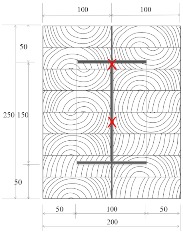	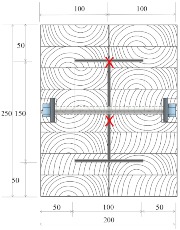
**DO**	**DB**	**CO**	**CB**
TSC with Douglas fir By glue connection	TSC with Douglas fir By dowel connection	TSC with Cedar By glue connection	TSC with Cedar By dowel connection

**Table 2:** Design charring rates (CRs) *β_0_* and *β_n_* of wood-based material.

Type of Timber	*β_0_* mm/min	*β_n_* mm/min
Glued laminated timber with a characteristic density of >290 kg/m^3^	0.65	0.7
Solid timber with a characteristic density of >290 kg/m^3^	0.65	0.8

**Table 3:** Failure modes.

Name	Glue			With 2 Bolts		
	Specimen 1	Specimen 2	Specimen 3	Specimen 1	Specimen 2	Specimen 3
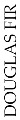	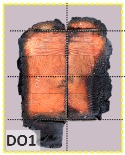	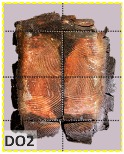	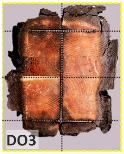	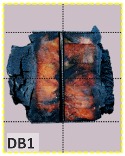	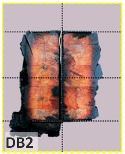	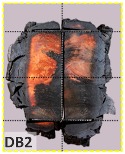
	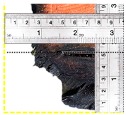	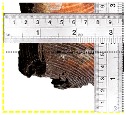	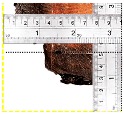	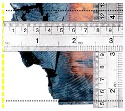	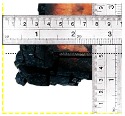	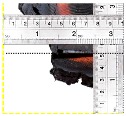
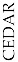	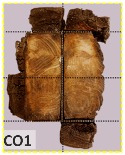	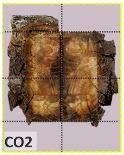	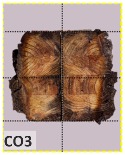	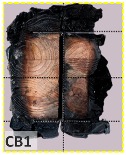	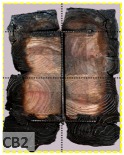	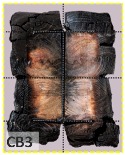
	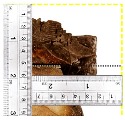	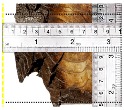	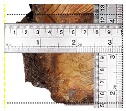	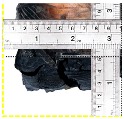	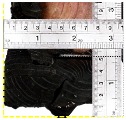	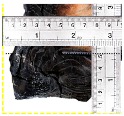

**Table 4:** Degradation of structure by charcoal area and highest temperature at flanges.

Case	Remain Area (mm^2^)	Area of Char (mm^2^)	Ratio of Char $A_{char}/A_{TSC}\;(\%)$	Ratio of Char $A_{char}/A_{extra\;layer\;}(\%)$	Highest Temperature at Flange (°C)
DO1	20,004	29,996	60.0	85.7	305
DO2	26,286	23,714	47.4	67.8	326
DO3	26,103	23,897	47.8	68.3	220
DB1	16,893	33,107	66.2	94.6	389
DB2	17,906	32,094	64.2	91.7	--
DB3	20,775	29,225	58.5	83.5	213
CO1	20,681	29,319	58.6	83.8	417
CO2	21,009	28,991	58.0	82.8	170
CO3	21,549	28,451	56.9	81.3	486
CB1	17,197	32,803	65.6	93.7	367
CB2	24,301	25,699	51.4	73.4	363
CB3	19,513	30,487	61.0	87.1	343

**Table 5:** Failure modes related to temperature and char properties.

Case	Highest Temperature at Flanges	Damage at the Web	Damage at the Flange	Failure Modes
DO1	*t* > 300 °C (high)	*d_char,max_ >* 5 cm (exceed)	*d_char,max_ >* 5 cm (exceed)	Failed
DO2	*t* > 300 °C (high)	*d_char,max_ <* 5 cm (within)	*d_char,max_ <* 5 cm (within)	Failed
DO3	*t* < 300 °C (low)	*d_char,max_ <* 5 cm (within)	*d_char,max_ <* 5 cm (within)	Satisfied
DB1	*t* > 350 °C (very high)	*d_char,max_ >* 5 cm (exceed)	*d_char,max_ >* 5 cm (exceed)	Failed
DB2	-----	*d_char,max_ >* 5 cm (exceed)	*d_char,max_ >* 5 cm (exceed)	Failed
DB3	*t* < 300 °C (low)	*d_char,max_ <* 5 cm (within)	*d_char,max_ <* 5 cm (within)	Satisfied
CO1	*t* > 350 °C (very high)	*d_char,max_ <* 5 cm (within)	*d_char,max_ >* 5 cm (exceed)	Failed
CO2	*t* < 300 °C (low)	*d_char,max_ <* 5 cm (within)	*d_char,max_ <* 5 cm (within)	Satisfied
CO3	*t* > 350 °C (very high)	*d_char,max_ >* 5 cm (exceed)	*d_char,max_ >* 5 cm (exceed)	Failed
CB1	*t* > 350 °C (very high)	*d_char,max_ >* 5 cm (exceed)	*d_char,max_ >* 5 cm (exceed)	Failed
CB2	*t* > 350 °C (very high)	*d_char,max_ >* 5 cm (exceed)	*d_char,max_ >* 5 cm (exceed)	Failed
CB3	*t* > 300 °C (high)	*d_char,max_ <* 5 cm (within)	*d_char,max_ <* 5 cm (within)	Failed

**Table 6:** Char depth (CD) and charring rate (CR) comparison based on methods.

Case	Average Charring Area Model		Specified Model (Euro Code 5)		Maximum Charring Depth Model	
	*d_char,average_* (mm)	*β_(average)_* (mm/min)	*d_char,n_* (mm)	*β_n_* (mm/min)	*d_char,max_* (mm)	*β_TSC(max)_* (mm/min)
DO1	40.69	0.68	42	0.7	60	1.00
DO2	30.48	0.53	42	0.7	44	0.73
DO3	30.76	0.55	42	0.7	43	0.72
DB1	46.32	0.77	42	0.7	54	0.90
DB2	44.44	0.74	42	0.7	56	0.93
DB3	39.36	0.66	42	0.7	50	0.85
CO1	39.52	0.66	42	0.7	53	0.88
CO2	38.96	0.65	42	0.7	50	0.83
CO3	38.05	0.61	42	0.7	52	0.88
CB1	45.75	0.74	42	0.7	56	0.93
CB2	33.56	0.60	42	0.7	59	0.98
CB3	41.55	0.69	42	0.7	51	0.85

**Table 7:** CD and required effective cross-section for timber within 1 h of burning.

Case	Specified Model (EU5) *d_char,n_* (mm)	Efficient Cross-Section (EU5) *d_ef_* (mm)	Average Charring Area Model *d_char,average_* (mm)	Max. Charring Depth Model *d_char,max_* (mm)
DO1	42	49	40.69	60
DO2	42	49	30.48	44
DO3	42	49	30.76	43
DB1	42	49	46.32	54
DB2	42	49	44.44	56
DB3	42	49	39.36	50
CO1	42	49	39.52	53
CO2	42	49	38.96	50
CO3	42	49	38.05	52
CB1	42	49	45.75	56
CB2	42	49	33.56	59
CB3	42	49	41.55	51

**Table 8:** Table of CRs generated by type of TSC.

Case	Average Charring Area Model (mm/min)	Specified Model (EU5) (mm/min)	White’s Model (mm/min)	Max. Charring Depth Model (mm/min)
DO	0.58	0.7	0.654	0.82
DB	0.72	0.7	0.654	0.89
CO	0.64	0.7	0.654	0.86
CB	0.68	0.7	0.654	0.92

**Table 9:** Table of char map area.

Name	Glue (Without Bolts)			With 2 Bolts		
	Specimen 1	Specimen 2	Specimen 3	Specimen 4	Specimen 5	Specimen 6
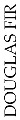	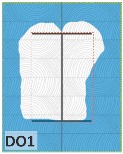	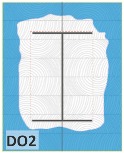	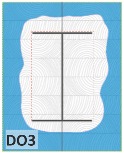	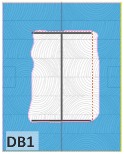	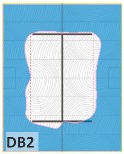	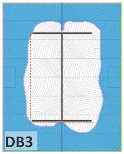
temp	305 °C	326 °C	220.4 °C	388.6 °C	--	212.7 °C
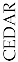	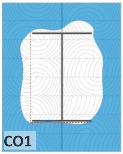	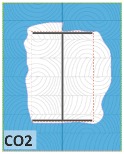	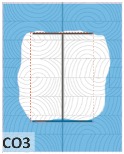	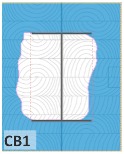	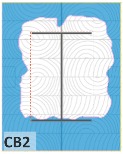	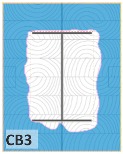
temp	416.8 °C	170.1 °C	486 °C	366.9 °C	362.8 °C	343.1 °C
